# An Arabidopsis ATPase gene involved in nematode-induced syncytium development and abiotic stress responses

**DOI:** 10.1111/tpj.12170

**Published:** 2013-03-08

**Authors:** Muhammad Amjad Ali, Stephan Plattner, Zoran Radakovic, Krzysztof Wieczorek, Abdelnaser Elashry, Florian MW Grundler, Moritz Ammelburg, Shahid Siddique, Holger Bohlmann

**Affiliations:** 1 Division of Plant Protection, Department of Crop Sciences, University of Natural Resources and Life Sciences Vienna, Universitäts- und Forschungszentrum TullnKonrad Lorenz Straße 24, Tulln, 3430, Austria; 2 Department of Molecular Phytomedicine, Institut für Nutzpflanzenwissenschaften und Ressourcenschutz, University of BonnBonn, 53115, Germany; 3 Department 1, Protein Evolution, Max Planck Institute for Developmental BiologySpemannstraße 35, Tübingen, 72076, Germany

**Keywords:** *Arabidopsis thaliana*, *Heterodera schachtii*, syncytium, AAA+ ATPase, GUS, amiRNA, abiotic stress, DAA1

## Abstract

The beet cyst nematode *Heterodera schachtii* induces syncytia in the roots of *Arabidopsis thaliana*, which are its only nutrient source. One gene, *At1g64110*, that is strongly up-regulated in syncytia as shown by RT-PCR, quantitative RT-PCR, *in situ* RT-PCR and promoter::GUS lines, encodes an AAA+-type ATPase. Expression of two related genes in syncytia, *At4g28000* and *At5g52882*, was not detected or not different from control root segments. Using amiRNA lines and T-DNA mutants, we show that *At1g64110* is important for syncytium and nematode development. *At1g64110* was also inducible by wounding, jasmonic acid, salicylic acid, heat and cold, as well as drought, sodium chloride, abscisic acid and mannitol, indicating involvement of this gene in abiotic stress responses. We confirmed this using two T-DNA mutants that were more sensitive to abscisic acid and sodium chloride during seed germination and root growth. These mutants also developed significantly smaller roots in response to abscisic acid and sodium chloride. An *in silico* analysis showed that ATPase At1g64110 (and also At4g28000 and At5g52882) belong to the ‘meiotic clade’ of AAA proteins that includes proteins such as Vps4, katanin, spastin and MSP1.

## Introduction

Nematodes are a large group of animals that include free-living and parasitic species of animals, humans and plants. Plant pathogenic nematodes parasitize a large variety of plant species, especially the roots, and cause serious damage to crop plants. Among the economically important pathogens are two main groups with a sedentary lifestyle. They induce a feeding site within the plant root that consists of several giant cells in case of root-knot nematodes (genus *Meloidogyne*) or a syncytium in case of cyst nematodes (genera *Heterodera* and *Globodera*). These feeding sites are the sole source of nutrients throughout the whole life of these sedentary nematodes (Gheysen and Mitchum, 2011).

Cyst nematodes enter the plant roots as second-stage juveniles (J2). They select a single root cell (initial syncytial cell) within the central cylinder, and induce a syncytium that expands by incorporating up to a few hundred neighbouring cells by partial cell-wall dissolution. Adult male cyst nematodes only feed from the syncytium until their third moult, and then leave their feeding site to mate with females. Female cyst nematodes never leave their feeding site and continue to feed after fertilization. They produce several hundred eggs that remain within their enlarged bodies, which subsequently harden to form cysts, which protect the eggs until infective J2 larvae hatch again under favourable conditions (Sobczak and Golinowski, 2011).

Development of the syncytium from the initial syncytial cell requires partial cell-wall dissolutions to neighbouring cells, and is probably initiated by nematode secretions that are delivered into the plant through the nematode stylet. The development of syncytia requires coordinated expression of a variety of plant genes, including expansins, cellulases and pectinases that are important for the degradation of cell walls, leading to incorporation of new cells into the growing syncytium (Goellner *et al*., 2001; Wieczorek *et al*., 2006, 2008). Syncytial cell walls are not only partially degraded but also undergo modifications that require the synthesis of new cell-wall polysaccharides. Cell-wall ingrowths that are thought to be important for the transport of water and solutes are produced at the interface between syncytia and xylem vessels in syncytia associated with female nematodes (Jones and Northcote, 1972; Siddique *et al*., 2012), and the outer cell walls of the syncytium are strengthened. The cells of the syncytium undergo drastic changes in ultrastructure and activity. The large central vacuole is fragmented into many small vacuoles, and the nuclei and nucleoli are enlarged due to endo-reduplication of DNA (Niebel *et al*., 1996). Syncytia show high metabolic activity, evident from proliferation of the endoplasmic reticulum and accumulation of ribosomes and mitochondria in a dense granular cytoplasm (Golinowski *et al*., 1996; Sobczak *et al*., 1997). At the transcriptome level, preferential up-regulation of GO categories related to metabolic activity was found (Szakasits *et al*., 2009). The nematodes withdraw solutes and nutrients from the syncytium through a feeding tube formed at the tip of their stylet, thus creating a strong sink within the plant. The feeding tube also acts as a molecular sieve, such that only molecules up to 20–30 kDa are taken up (Böckenhoff and Grundler, 1994; Urwin *et al*., 1998).

The sugar beet cyst nematode *Heterodera schachtii* completes its lifecycle on *Arabidopsis thaliana* roots *in vitro* within 6 weeks (Sijmons *et al*., 1991). This interaction has been established as a model system due to the fact that the translucent Arabidopsis roots facilitate microscopic study of the development of this and other nematode species inside living roots (Wyss and Grundler, 1992). We have recently performed a transcriptome analysis of syncytia induced by *H. schachtii* in Arabidopsis roots that revealed that, of 21 138 genes represented on the Affymetrix GeneChip, 18.4% had a higher expression level and 15.8% had a lower expression level in syncytia compared with control roots (Szakasits *et al*., 2009).

One of the most strongly up-regulated genes (*At1g64110*, Table S1) was found to encode an AAA+-type ATPase. This gene was also found to be regulated by the R2R3 MYB transcription factor DUO1 and induced in sperm cells (Borg *et al*., 2011). It was therefore named *DAA1* (DUO1-activated ATPase1). AAA+-type ATPases form a large superfamily of proteins that contain a P-loop NTPase domain. They have a diverse range of functions, for example as subunits of proteases or as molecular chaperones involved in the unfolding and disaggregation of macromolecules (Iyer *et al*., 2004; Ammelburg *et al*., 2006). Considering the rearrangements that take place in the cells that are incorporated into the syncytium, it is clear that such activities are needed for the development of syncytia. We therefore studied expression of the *At1g64110*/*DAA1* gene in detail, and show its importance for the biotic interaction with the beet cyst nematode *H. schachtii* and also for abiotic stress responses.

## Results

### Expression of ORTHO000440 genes in Arabidopsis

**Figure 1:**
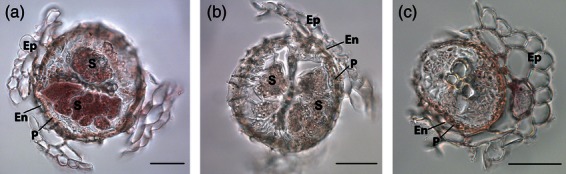
*In situ* RT-PCR of *At1g64110* expression in 15 dpi syncytia and uninfected roots. (a) Strong signal for *At1g64110* mRNA in the syncytium. In addition, staining is visible in pericycle cells. (b) Control reaction performed without *Taq* polymerase on a section of 15 dpi syncytium. Staining is not visible in either the feeding site or surrounding cells. (c) Control reaction on a root section above the syncytium showing signals for *At1g64110* in pericycle cells. S, syncytium; Ep, epidermis; En, endodermis; P, pericycle. Scale bars = 50 μm.

The *At1g64110*/*DAA1* gene belongs to a small sub-family of three very similar genes in *A. thaliana* and *Arabidopsis lyrata*, designated ORTHO000440 by PLAZA (http://bioinformatics.psb.ugent.be/plaza/, Proost *et al*., 2009) within the gene family HOM000025. The two other genes in this sub-family are *At4g28000*, which is expressed at very low levels in roots and syncytia (Szakasits *et al*., 2009), and *At5g52882*, which is not represented on the Affymetrix Arabidopsis GeneChip (Table S1). We performed a semiquantitative RT-PCR using RNA isolated from syncytia cut from infected roots at 5 and 15 dpi (days post-inoculation). Corresponding root segments without nematode infection were used as a control (Figure S1). Expression of *At1g64110* and *At5g52882* was clearly detected. Expression of *At1g64110* was stronger in syncytia than in control root segments, but there was not much difference between syncytia and control root segments in terms of expression of *At5g52882*, confirming transcriptome data (Szakasits *et al.*, 2009). We then used quantitative RT-PCR (Table 1) to compare expression in 15 dpi syncytia cut from infected roots with that in control root segments without root tips (as used by Szakasits *et al.*, 2009). Expression of *At1g64110* was strongly up-regulated in syncytia, with a fold change of 97.2, but expression of *At5g52882* was not different in syncytia compared to control root segments (fold change of 0.95). In order to localize expression of *At1g64110* in and around the syncytium, we used *in situ* RT-PCR on sections from 15 dpi syncytia (Figure 1). A high level of *At1g64110* transcripts was clearly detected in syncytia. In uninfected root sections, expression was detected in pericycle cells but not in cells of the stele. No products were observed in control root sections without *Taq* polymerase.

**Table 1:** ORTHO000440 gene expression in syncytia (quantitative RT-PCR)

	ΔΔ*C*_t_ (log_2_)	Fold change
*At1g64110*	7.255	97.2
*At5g52882*	0.08	0.95

Expression in 15 dpi syncytia (cut from the roots) compared to control root segments comprising the elongation zone without root tips. Values are the means of three technical and three biological replicates.

**Figure 2:**
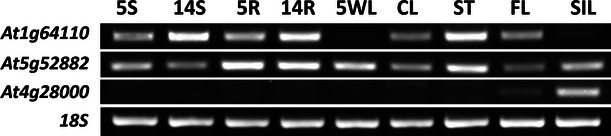
RT-PCR using RNA isolated from seedlings grown on MS medium (5S, 5-day-old shoots; 14S, 14-dayold shoots; 5R, 5-day-old roots; 14R, 14-day-old roots) or on soil (5WL, 5-week-old leaves; CL, cauline leaves, ST, stems; FL, flowers; SIL, siliques). Primers for the 18S gene were used for control reactions.

We performed RT-PCR for all three genes using RNA from various organs and growth stages (Figure 2). Expression of *At1g64110* and *At5g52882* was detected in most organs. *At1g64110* and *At5g52882* were expressed in the roots and shoots of 5- and 14-day-old seedlings. *At1g64110* was expressed in stems and flowers but not in siliques, whereas *At5g52882* was expressed in stems and flowers and also in siliques. Expression of *At4g28000* was only detected in siliques and weakly in flowers. The results for *At1g64110* and *At4g28000* are in line with GeneChip data available at Genevestigator (https://www.genevestigator.com/, Zimmermann *et al*., 2004) (Figure S2).

### GUS expression analysis of *At1g64110*

**Figure 3:**
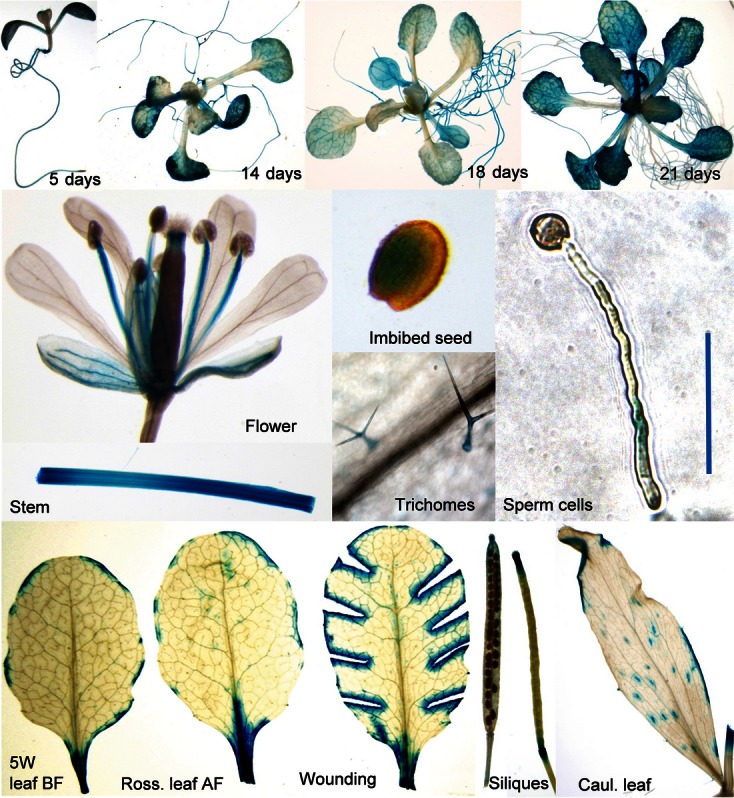
GUS expression at various growth stages (5-, 14-, 18- and 21-day-old seedlings) and in various organs of a representative *At1g64110* promoter::GUS line. BF, before flowering; AF, after flowering. Scale bar = 100 μm.

Of the three genes of this sub-family, only *At1g64110* expression was strongly induced in syncytia. We therefore created promoter::GUS lines to further study its expression. A representative line was selected, and the general GUS expression pattern was assessed (Figure 3). Seedlings showed staining in the roots and the shoots. Older rosette leaves were not stained except for trichomes, with some staining at the edge of the leaves. We also noted staining at the petiole where the leaves were cut from the plant, suggesting induction of the gene by wounding. We confirmed this by cutting the leaves, which led to staining at the cut sites. Cauline leaves also showed some staining at the edges, and also strong staining in the trichomes. Stems were also stained, and we observed staining of the filaments of the anthers and the veins of the sepals in flowers. Some staining was also observed at the base of the stigma, and also in young siliques but not old siliques. Young siliques were also stained at the abscission zone, and seeds were only stained after imbibition. Lastly, GUS expression was observed in sperm cells after artificial germination of pollen grains.

**Figure 4:**
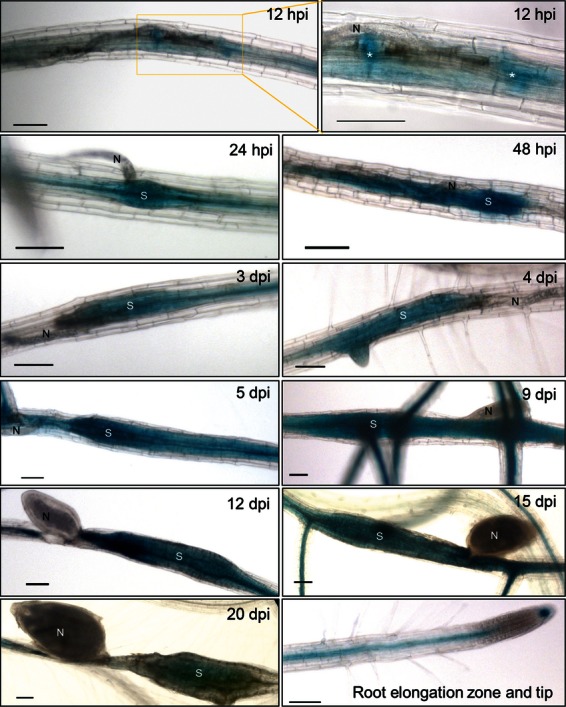
GUS expression in syncytia of a representative *At1g64110* promoter::GUS line at various times after inoculation. There was strong expression of GUS throughout the development of nematode-induced syncytia, starting from cell differentiation for syncytia formation (12 hpi) up to mature syncytia (15 dpi), and decreasing at 20 dpi. Asterisks indicate cells differentiating into syncytia; N, nematode; S, syncytium. Scale bar = 100 μm. The last image shows the root elongation zone and root tip from a non-infected plant.

The promoter::GUS line was used to determine the expression of *At1g64110* during syncytium development (Figure 4). Even at 12 hpi (hours post-inoculation), faint GUS staining was visible that appeared to be associated with cells that are then incorporated into the developing syncytium. Clear GUS staining of the syncytium was detected at 24 hpi, and at all time points thereafter (48 hpi, 3 dpi, 4 dpi, 5 dpi, 9 dpi and 12 dpi), up to 15 dpi. At 20 dpi, the GUS staining was much weaker. Uninfected seedlings showed GUS staining in roots and shoots at all time points (Figure S3) that decreased in older seedlings, and there was usually no staining in roots by 22 days.

### Down-regulation of *At1g64110* enhances resistance against *H. schachtii*

**Figure 5:**
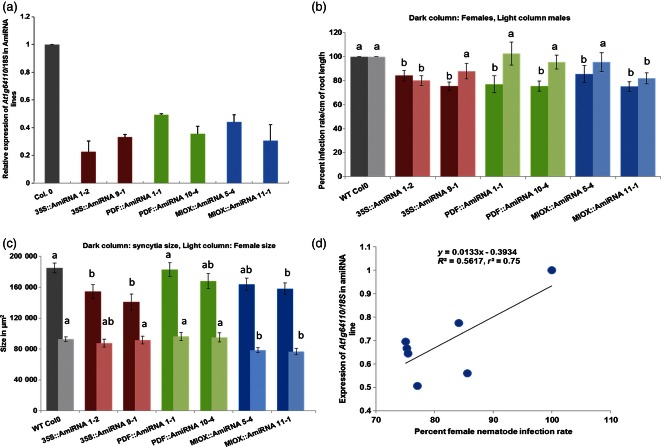
Down-regulation of *At1g46110* expression enhances resistance against *H*. *schachtii*. Three promoters (35S, *Pdf2.1* and *Miox5*) were used to drive expression of an artificial *At1g46110* miRNA gene. (a) *At1g46110* expression in miRNA lines measured by quantitative RT-PCR relative to expression of the 18S gene in syncytia cut from infected roots at 15 dpi. Values are means ± SEM for three biological and three technical replicates. (b) Infection rate per cm of root length calculated at 15 dpi for male and female nematodes. (c) Size of female syncytia and female nematodes at 14 dpi (*n* = 24). In (b) and (c), data were analysed for significant differences using ANOVA (*P* < 0.05) and LSD. Values are means ± SE. Different letters indicate differences that are significant at *P* < 0.05. (d) Correlation of infection rate with expression of *At1g64110* in miRNA lines with various promoters.

The strong up-regulation of *At1g64110* in syncytia indicated an important function of this gene for syncytium development. We tested this by producing artificial micro RNA (amiRNA) lines to down-regulate the gene using three promoters: the CaMV 35S promoter, the *Pdf2.1* promoter (Siddique *et al*., 2011), and the *Miox5* promoter (Siddique *et al*., 2009). The 35S promoter has been reported to be only active in younger syncytia and down-regulated in old syncytia (Urwin *et al*., 1997; Goverse *et al*., 1998), whereas both *Pdf2.1* and *Miox5* have been shown to be strongly up-regulated in syncytia. For each promoter, we selected two independent homozygous lines with at least 50% down-regulation of the *At1g64110* gene (Figure 5a). We performed infection assays for all lines, and found that the number of female nematodes was significantly reduced in each line but the number of males was not significantly different (Figure 5b). Contrasting results were found for the size of syncytia and females in these lines (Figure 5c). In the 35S promoter lines, the size of the syncytia was significantly reduced but not the size of females. In the *Miox5* promoter lines, the sizes of both females and syncytia were reduced. No differences were found for the *Pdf2.1* promoter miRNA lines. A strong correlation (*r*^2^ = 0.82) was found between the nematode infection rate and silencing of *At1g64110* in 11 tested amiRNA lines with the various promoters (Figure 5d). A low positive correlation was found between syncytium size and female nematode size (*r*^2^ = 0.73 and *r*^2^ = 0.32, respectively) and silencing of *At1g64110* in amiRNA lines (Figure S4)

### *At1g64110* and *At5g52882* are important for syncytium and nematode development

**Figure 6:**
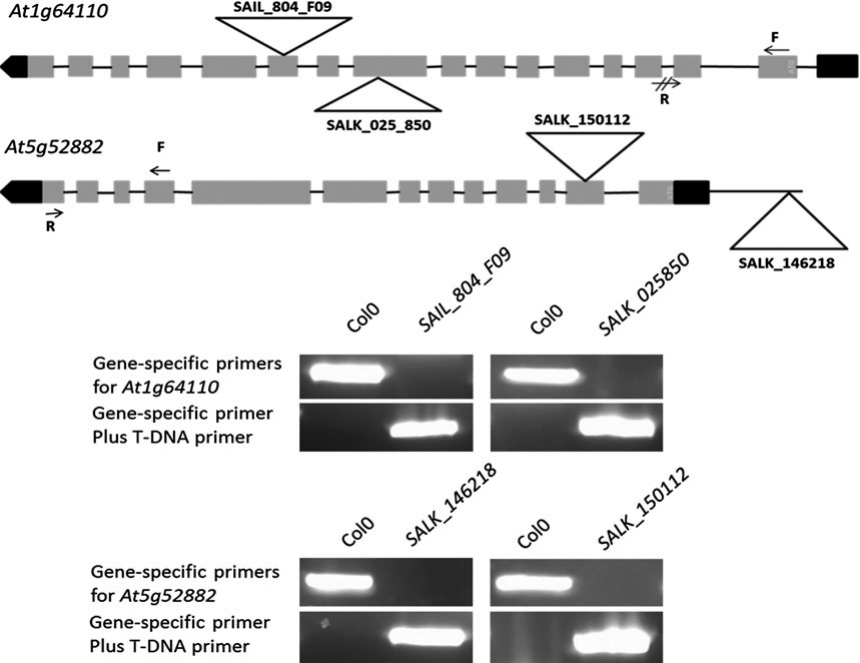
T-DNA mutants for *At1g46110* and *At5g52882*. The intron/exon structure and the site of the T-DNA insertion are shown for both genes. PCR using gene-specific primers and the T-DNA primer confirmed homozygous mutants.

**Figure 7:**
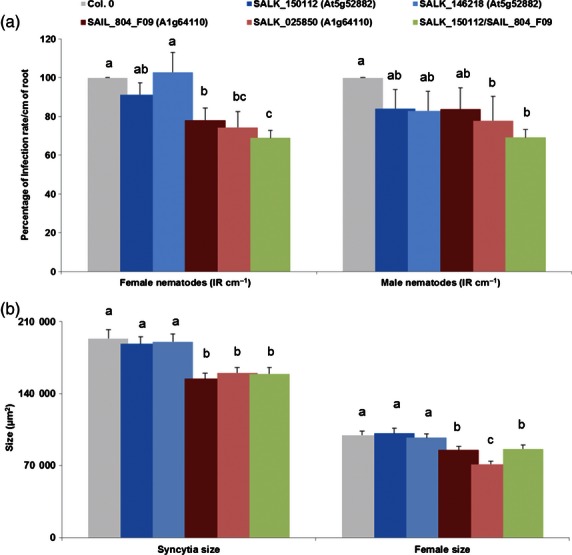
Infection assays for knockout mutants of *At1g64110* (SAIL_804_F09 and SALK_025850), *At5g52882* (SALK_150112 and SALK_146218) and a double mutant (SALK_150112/SAIL_804_F09) compared with Col wild-type plants. (a) Infection rate per cm root length calculated at 15 dpi, with wild-type set as 100%, for female and male nematodes. (b) Size of female syncytia and female nematodes at 14 dpi (*n* = 30). Different letters indicate differences that are significant at *P* < 0.05 (anova and LSD). Values are means ± SE.

**Figure 8:**
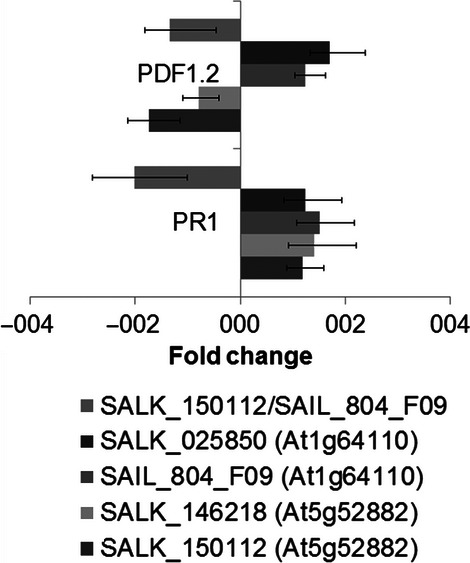
Expression of defence marker genes in *At1g46110* and *At5g52882* T-DNA mutants. Relative expression of *Pdf1.2* and *PR1* measured by quantitative RT-PCR in seedlings of knockout mutants compared with Col wild-type plants. There was no change in expression of *Pdf1.2* and *PR1* in any of the mutants. Values are means ± SEM for three technical and three biological replicates.

We isolated homozygous T-DNA mutants for the *At1g64110* gene (two mutants with insertions in exons) and also for the *At5g52882* gene (one mutant with an insertion in an exon and one mutant with an insertion in the promoter), which was also found to be expressed in syncytia (Figure 6). In addition to these single mutants, we constructed a double mutant by crossing the lines SAIL_804_F09 *(At1g64110*) and SALK_150112 (*At5g52882*). PCR was used to confirm homozygosity of all mutants. We excluded the *At4g28000* gene from this analysis because it is not expressed in syncytia. Except for the SALK_146218 mutation, which is located in the promoter, the other three T-DNA lines were knockout mutants, as shown by quantitative RT-PCR. No expression was detected for *At1g64110* in either mutant line (SALK_025850 and SAIL_804_F09). For *At5g52882*, no expression was detected in SALK_150112; however, normal expression was detected in SALK_146218 (Table 2). We analysed the seedling root phenotype of these mutants. There was a significant decrease in root area and length for SALK_146218 (*At5g52882*) compared to wild-type seedlings. For all other mutants, including the double mutant, no differences were found (Figure S5). We tested the susceptibility of the mutants (single and double) and compared them with Col wild-type plants (Figure 7). Single mutants of gene *At5g52882* had the least effect. The number of female and male nematodes was not significantly different from wild-type, and also the size of syncytia and of female nematodes was the same. However, both single mutants of gene *At1g64110* were clearly more resistant than wild-type. They supported fewer females and males, and also syncytia and females were smaller than those from wild-type plants. The double mutant was not significantly different from the *At1g64110* single mutants. We also analysed expression of the defence marker genes *Pdf1.2* (Thomma *et al*., 1998) and *PR1* (Uknes *et al*., 1992) in the mutants (Figure 8). We found no difference in expression in seedlings of any of the mutants compared to wild-type seedlings.

**Table 2:** Expression of *At1g64110* and *At5g52882* in T-DNA mutants

Locus	T-DNA mutant lines	Expression
*At1g64110*	SAIL_804_F09	ND
	SALK_025850	ND
*At5g52882*	SALK_150112	ND
	SALK_146218	+

ND, not detected; +, normal expression.

RNA was extracted from 7-day-old seedlings to detect expression of *At1g64110* and *At5g52882* in corresponding T-DNA mutant lines by quantitative RT-PCR.

### *At1g64110* is involved in the response to abiotic stress

**Figure 9:**
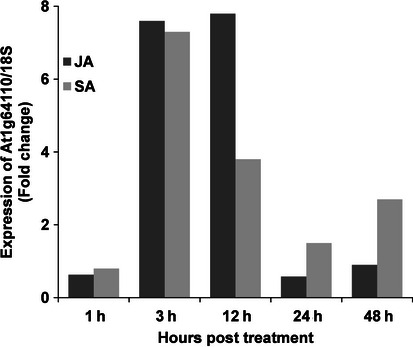
Relative expression of the *At1g64110* gene in response to MeJA (100 μM) and SA (1 mm). The graph shows the results for one of two experiments with similar results.

The finding that *At1g64110* was induced by wounding prompted us to test its induction by jasmonic acid (JA). We also included salicylic acid (SA) as it is commonly involved in resistance responses to biotrophic pathogens. Treatment with methyl jasmonic acid (MeJA) and SA was performed by infiltrating seedlings growing on MS medium. As shown in Figure 9, *At1g64110* expression was induced by JA, especially after 3 and 12 h. The strongest response to SA was found at 3 h, after which it declined, but increased again at 48 h.

**Figure 10:**
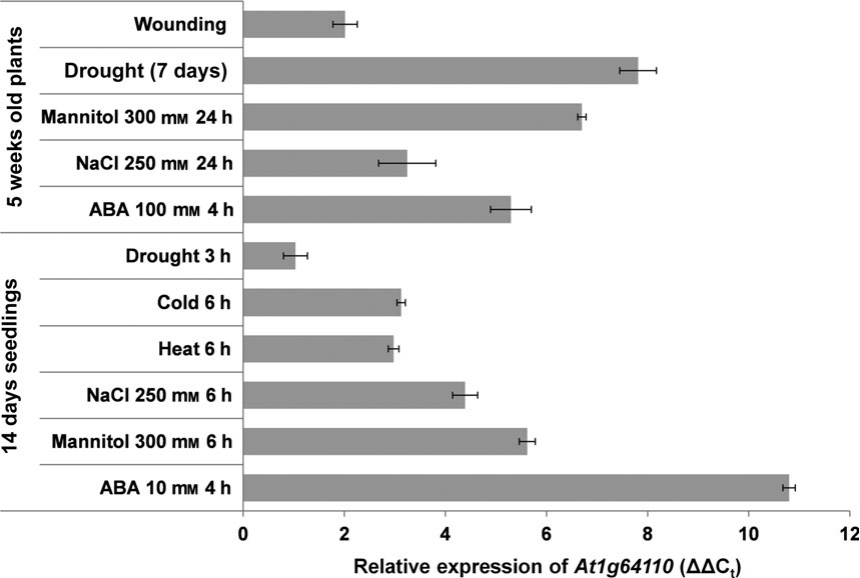
Relative expression of *At1g64110* in wild-type seedlings subjected to various stresses at early (14-day-old seedlings on MS medium) and late (5-week-old rosette leaves on soil) developmental stages compared to control plants as measured by quantitative RT-PCR. The experiment was repeated twice with three technical replicates, and the data are shown as 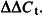 The values are means ± SE.

**Figure 11:**
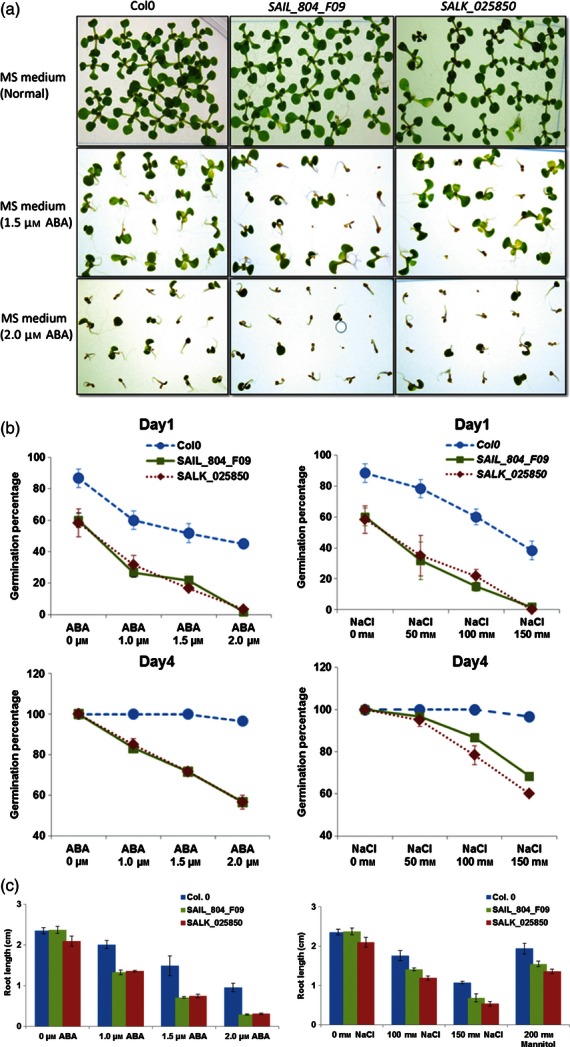
Physiological responses of *At1g64110* mutants (SAIL_804_F09 and SALK_025850). (a) Germination of wild-type and two mutants grown for 10 days on MS agar plates containing various ABA concentrations. (b) Effects of ABA and NaCl on germination at day 1 and day 4. (c) Effects of NaCl and mannitol on the root length of seedlings grown on MS agar plates. Data are means ± SE of three experiments with *n* = 20 (germination) and *n* = 6 (root length).

We further performed quantitative RT-PCR to study expression of the ATPase gene *At1g64110* in response to various abiotic stress conditions, including wounding, abscisic acid (ABA), cold, heat, drought, salt and osmotic stresses at the 14-day seedling stage and 5-week rosette stage. The *At1g64110* gene was highly induced in response to these various abiotic stresses (Figure 10). However, the induction was more profound in response to ABA, mannitol and NaCl at the early seedling stage. To test the importance of this gene in the response to these physiological stresses, we produced synchronized seed populations harvested from plants grown at the same time. These seeds of the same age from control (Col) and two T-DNA insertion knockout mutants of *At1g64110* were plated on Murashige and Skoog (MS) medium in the presence of various concentrations of ABA, NaCl or mannitol, and stratified for 3 days at 4°C. The germination percentage was scored, and seedling root lengths were measured. The results revealed that knockout mutants showed slow germination under control conditions (at day 1 but not day 4), and also had increased sensitivity to ABA and NaCl during seed germination and root growth (Figure 11a,b). The mutants also developed significantly smaller roots in response to ABA, NaCl and mannitol (Figure 11c). Together, these experiments indicate that *At1g64110* knockout mutants are impaired in germination and root growth in response to ABA, NaCl and mannitol, which is indicative of stress sensitivity and the role of this gene in stress tolerance.

### *At1g64110* is not regulated by DUO1 in syncytia

The induction of *At1g64110*/*DAA1* in sperm cells is regulated by the R2R3 MYB transcription factor DUO1 (Borg *et al*., 2011). To test whether DUO1, which is not included on the Affymetrix GeneChip, may also be responsible for the strong induction of *At1g64110*/*DAA1* in syncytia, we performed a quantitative RT-PCR experiment. Syncytia were cut out from infected roots and compared to uninfected root segments. No difference was found for the expression of *DUO1* in 5 and 15 dpi syncytia compared to roots, indicating that this transcription factor is probably not responsible for the strong expression of *At1g64110*/*DAA1* in syncytia (Table 3).

**Table 3:** Expression of *DUO1* in 5 and 15 dpi syncytia (quantitative RT-PCR)

Time point	ΔΔ*C*_t_ (log_2_)	Fold change
5 dpi	−0.75	−1.68
15 dpi	0.24	1.18

Syncytia were cut from the roots, and expression of *DUO1* was compared to that in control root segments. Values are the means of three technical and three biological replicates.

### Sequence analysis of ORTHO000440 proteins

**Figure 12:**
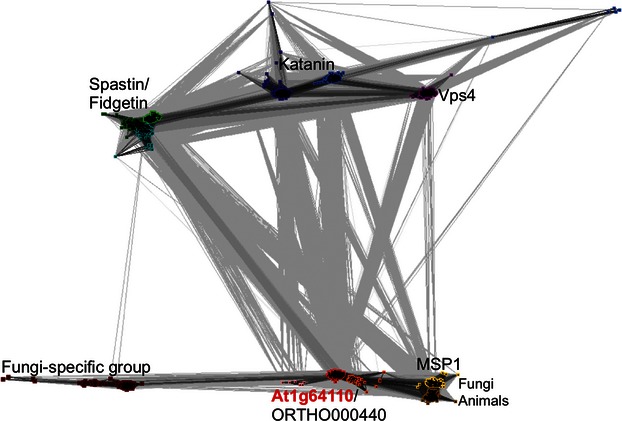
Cluster analysis of the ‘meiotic clade’ of AAA proteins. The cluster map illustrates the relationships of proteins belonging to the meiotic clade of AAA proteins (Frickey and Lupas, 2004b). The map contains 1734 sequences, each of which is represented by a dot. Pairwise similarities of the conserved AAA domain of all sequences were calculated using BLAST (Altschul *et al*., 1997). Sequences were clustered using CLANS (Frickey and Lupas, 2004a) using similarities with *P* values < 1.0e-50. Darker lines between two dots indicate lower BLAST *P* values. The At1g64110/ORTHO000440 cluster (red dots) shows the tightest connections to MSP1 proteins from fungi and metazoa (yellow dots). However, the Spastin (green), Katanin (blue) and Vps4 (purple) clusters also show strong similarities to the At1g64110/ORTHO000440 (red) group.

The AAA+ ATPase proteins studied here are very similar at the protein level as shown by a sequence alignment (Figure S6). They do not contain a signal sequence (Petersen *et al.*, 2011) but do contain a transmembrane helix at the N-terminus (Figure S7). They contain two AAA domains, but only the second one appears to be functional as it comprises the catalytical Walker A and B sequences (Walker et al., 1982), which are missing from the first domain. Furthermore, a C-terminal extension, similar to the one found in Vps4 (Babst et al., 1998) and presumably important for oligomerization, was detected. Sequence comparison with other ATPases positioned the ORTHO000440 proteins within the central cluster of the AAA family, within the AAA+ superfamily (Ammelburg et al., 2006). Further sub-typing allowed assignment of At1g64110 to the ‘meiotic clade’ of AAA proteins (Frickey and Lupas, 2004b), containing AAA proteins such as Vps4, katanin, spastin and MSP1 (Figure 12). The most pronounced similarity detected is to the membrane-bound MSP1 (mitochondrial sorting of proteins 1) proteins of fungi and metazoa, with BLAST *P* values as low as 1.0e-70.

## Discussion

### Expression of ATPase genes in response to nematode infection

The ATPase gene *At1g64110* was among the most strongly up-regulated genes found in a transcriptome analysis of syncytia induced by the beet cyst nematode *H. schachtii* in Arabidopsis roots (Szakasits *et al*., 2009). Here we confirmed the expression in syncytia using RT-PCR, *in situ* RT-PCR and GUS analysis. Arabidopsis contains two other genes that are related to *At1g64110*: *At4g28000* and *At5g52882*. *At4g28000* was expressed at a very low level in syncytia and control root segments (Szakasits *et al*., 2009), and was only detected in the present study in flowers and siliques using RT-PCR; thus we have excluded this gene from further experiments. *At5g52882* is not represented on the Affymetrix Arabidopsis GeneChip, and therefore no information was available about its expression. We therefore tested the expression of this gene in syncytia using RT-PCR and quantitative RT-PCR. Expression was clearly detected in syncytia and roots, but was not up-regulated in syncytia, in contrast to *At1g64110* expression.

### *At1g64110* is expressed in sperm cells

It has been shown that *At1g64110* is expressed in sperm cells, and that this expression is dependent on the R2R3 MYB transcription factor DUO1 (Borg *et al*., 2011). We confirmed the expression of *At1g64110* in sperm cells using our promoter::GUS line. Unfortunately, *DUO1* (*At3g60460*) is not represented on the Arabidopsis GeneChip, and its expression in syncytia was therefore not known. We therefore used quantitative RT-PCR to show that expression of *DUO1* was not induced in syncytia compared to uninfected control root segments. Furthermore, examination of the expression level in syncytia (Szakasits *et al*., 2009) of DUO1 target genes in sperm cells (Borg *et al*., 2011) revealed that, other than *At1g64110*, only one gene (*DAN1*) was up-regulated in syncytia compared to control root segments and *DAW1* was down-regulated. All other DUO1 target genes were expressed at a very low level in roots and syncytia (Table S3). Thus, it is unlikely that DUO1 is responsible for the strong up-regulation of *At1g64110* in syncytia.

### ATPase genes are important for syncytium and nematode development

The strong up-regulation of *At1g64110* in syncytia suggested an important function of this gene for syncytium and nematode development. We confirmed this assumption using amiRNA lines and T-DNA knockout mutants. Expression of the amiRNA gene was driven by one of three promoters, and all lines achieved at least 50% reduction of the transcript level in syncytia cut from infected Arabidopsis roots. All lines showed an effect, and especially the number of females developing on these roots was reduced. The reduction in syncytium size in some amiRNA lines and in the *At1g64110* T-DNA mutants showed that *At1g64110* is important for syncytium development. The smaller syncytia provide fewer nutrients for the nematodes, and thus influence the development of nematodes. The observed effects were stronger with the two T-DNA mutants that were used, probably due to the fact that the miRNA lines still had a significant level of *At1g64110* transcripts while both *At1g64110* T-DNA mutants were knockout mutants. The effect of the *At5g52882* mutants was less pronounced. An *At1g64110* and *At5g52882* double mutant showed an additive effect, especially on the number of female nematodes developing on the roots. These data indicate that both proteins (and perhaps also At4g28000) have the same function, and that a high level of these proteins is needed for fully functional syncytia.

The ATPase genes discussed here may also be important for the development of clubroot disease. The pathogen *Plasmodiophora brassiceae* induces formation of galls in the roots of *Brassicaceae*, including Arabidopsis, which are a severe nutrient sink for the plant, similar to the syncytia induced by cyst nematodes. In a transcriptome analysis of clubroots of Arabidopsis roots, it was found that expression of *At1g64110* was almost 54-fold up-regulated (Siemens *et al*., 2006). It may be interesting to determine whether down-regulation of this gene led to enhanced resistance against *Plasmodiophora brassiceae*. Furthermore, induction of the gene by SA indicates that it may also be involved in resistance responses to other pathogens.

### The ATPase gene *At1g64110* is involved in abiotic stress responses

We found that *At1g64110* was inducible by MeJA and wounding, heat, cold and drought, as well as NaCl, ABA and mannitol. Involvement of the ATPase gene *At1g64110* in abiotic stress has been reported previously. Analysis of drought responses of *Boechera holboelli*, a relative of Arabidopsis, found that the *B. holboelli* homologue of *At1g64110* was up-regulated during drought responses (Knight *et al*., 2006). Drought responses in Arabidopsis are regulated by the plasma membrane histidine kinase ATHK1, and *At1g64110* is one of the genes that was induced in ATHK1 over-expression lines and down-regulated in an *athk1* mutant (Wohlbach *et al*., 2008). However, expression of *ATHK1* in syncytia was rather low, and was not different from that in control root segments (Szakasits *et al*., 2009), which indicates that the expression of *At1g64110* in syncytia may not be regulated by ATHK1. Furthermore, among the genes reported by Wohlbach *et al*. (2008) as up-regulated by *ATHK1*, only 11 were up-regulated in syncytia compared to control root segments (Szakasits *et al*., 2009), while three were down-regulated and the majority (24) showed the same expression in syncytia and roots (Table S4; data from Szakasits *et al*., 2009). Another transcription factor that was shown to regulate the expression of *At1g64110* was AREB1, a leucine zipper protein encoded by *At1g45249* that binds to the ABRE motif in the promoter region of ABA-inducible genes (Fujita *et al*., 2005). Again, most of the genes that were found to be up-regulated in transgenic lines over-expressing a constitutive active form of AREB1 (AREB1ΔQT) were not up-regulated in syncytia (Szakasits *et al*., 2009), indicating that AREB1 is also probably not involved in the strong up-regulation of *At1g64110* in syncytia (Table S5).

A proteomic study of brassinosteroid signal transduction in Arabidopsis using pre-fractionation and two-dimensional difference gel electrophoresis showed that expression of the ATPase-encoding gene *At1g64110* is induced in response to the brassinosteroid treatment (Tang *et al*., 2008). However, it is currently unknown whether brassinosteroids are involved in induction of *At1g64110* expression in syncytia.

The induction in response to various abiotic stresses indicated that *At1g64110* may be involved in the plant response to abiotic stresses. We tested this using two independent T-DNA mutants, and found that the gene plays an important role in germination and seedling root growth in response to ABA and NaCl. It may be interesting to extend these investigations to other stresses and to test whether the two other genes in this group (*At4g28000* and *At5g52882*) have the same effect.

### Function of ORTHO000440 ATPase genes

The three genes studied here belong to the sub-family designated ORTHO000440 by PLAZA (http://bioinformatics.psb.ugent.be/plaza/, Proost *et al*., 2009) within the gene family HOM000025, and encode typical ATPase proteins. Sequence comparison indicated that they belong to the AAA group within the AAA+ superfamily (Ammelburg *et al*., 2006).

Proteins of the meiotic clade of AAA proteins, such as Vps4, katanin and spastin, fulfil functions in membrane deformation and microtubule severing (Hill and Babst, 2012; Lumb *et al*., 2012). Furthermore, the yeast orthologue from the most similar sub-group, MSP1, has been shown to play a role in insertion of proteins into the inner mitochondrial membrane (Nakai *et al*., 1993). The rather close relationship of At1g64110 to these proteins implicates this plant-specific sub-group in the remodelling of membranes and shaping of their protein content. Such activities are relevant for processes such as formation of a syncytium or the response to stress, and provide a plausible explanation for the effects observed. To further explore the specific function of At1g64110 and related ATPases in Arabidopsis, it will be important to identify their subcellular location and to identify their interacting proteins, for example by using yeast two-hybrid screens or related methods.

## Experimental Procedures

### Plant cultivation and treatment

Arabidopsis seeds were surface-sterilized for 20 min in 6% w/v sodium hypochlorite, and subsequently washed three times with sterile water for 1 min at room temperature. Seedlings were grown in 9 cm Petri dishes on a modified Knop medium (Sijmons *et al*., 1991) supplemented with 2% sucrose under sterile conditions or on MS medium (Epple *et al*., 1995) in a growth chamber at 25°C under a 16 h light/8 h dark cycle.

Germination and growth assays of mutants were performed using age-matched seed populations of wild-type and mutants grown at the same time. For seed germination and root growth assays, seeds were sterilized and plated on 0.8% agar plates containing half-strength MS medium supplemented with various concentrations of ABA, NaCl or d-mannitol (all from Sigma-Aldrich; http://www.sigmaaldrich.com). A seed was considered as germinated when the radical protruded from the seed coat. Three plates each of 20 seeds per line were scored in germination assays. Three plates with six seeds per line were scored for root growth.

The expression of *At1g64110* in response to MeJA, SA, ABA, mannitol and NaCl was studied by infiltrating the solutions under vacuum into seedlings grown for 14 days on MS medium containing 3% sucrose (Epple *et al*., 1995). After infiltration, the seedlings were returned to the growth chamber, and samples were taken at various time points (1, 3, 12, 24 and 48 h). The fold change was determined by quantitative RT-PCR according to the 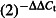 method (Livak and Schmittgen, 2001). The data were corrected by mock infiltration with tap water. Values are the means of two independent experiments (*n* = 2) and three technical replicates.

For treatment of older plants, we used 5-week-old rosette leaves from plants grown on soil. Wounding was performed using forceps, heat treatment was at 37°C, and cold treatment was at 4°C. For drought treatment, soil-grown plants were not watered for 7 days, while seedlings growing in Petri dishes on MS medium were treated by opening the plates and incubating them on a sterile bench. Untreated plants were used as a control for these treatments.

### Cloning of an amiRNA construct for *At1g64110*

Engineering of the precursor containing the artificial microRNA (amiRNA) was performed as described by Schwab *et al*. (2006). The primer sequences (Table S2) used to create the precursor of the artificial miRNA (amiRNA sequence: 5′-TAAACGTTTATGAAACTCGCC-3′) were generated using the web-based tool ‘Web microRNA Designer’ at http://wmd.weigelworld.org/cgi-bin/mirnatools.pl (Schwab *et al*., 2006).

The amiRNA-containing precursor was generated by overlapping PCR as described by Schwab *et al*. (2006). The first round amplified fragments (a) to (c). These were subsequently fused by PCR (d). Oligonucleotide primers I–IV were used to replace the miRNA319a region with the artificial sequence in plasmid pUCmi319a (see Methods S1). Primers A and B were based on the template plasmid sequence. Regeneration of functional miRNA precursors was achieved by combining PCR products A-IV, II-III and I-B in a single reaction with primers A and B.

### Arabidopsis transformation

Binary vectors were introduced into *Agrobacterium tumefaciens* GV3101 for transformation of Arabidopsis Col-0 plants by the floral-dip method as modified by Logemann *et al*. (2006). Transgenic plants were selected on MS medium containing 50 μg ml^−1^ kanamycin and 250 μg ml^−1^ timentin. Dry seeds were sprinkled onto the selection plates at approximately 25–35 seeds per cm^2^. The plates were incubated at 22°C with a photoperiod of 16 h light/8 h dark for the selection of kanamycin-resistant plants. Arabidopsis plants resistant to kanamycin were transferred to soil in a growth chamber to produce seeds.

### RNA isolation

Root segments containing syncytia were excised at 15 dpi and immediately frozen in liquid nitrogen. Control root segments were collected and frozen as described previously (Szakasits *et al*., 2009). Total RNA was isolated using an RNeasy plant mini kit (Qiagen; http://www.qiagen.com) according to the manufacturer's instructions, including DNase I digestion. The quality and quantity of the RNA was assessed using an Agilent 2100 bioanalyzer (Agilent Technologies; http://www.home.agilent.com). Reverse transcription was performed using SuperScript III reverse transcriptase (Invitrogen; http://www.invitrogen.com) and random primers [oligo(dN_6_)] according to the manufacturer's instructions.

### Semi-quantitative RT-PCR

RT-PCR was performed using RT-PCR Master Mix (USB; http://www.affymetrix.com/estore/browse/brand/usb/usb.jsp) according to the manufacturer's instructions. The primers used are shown in Table S2.

### Quantitative RT-PCR

Real-time RT-PCR was performed using an ABI PRISM 7300 sequence detector (Applied Biosystems; http://www.invitrogen.com/site/us/en/home/brands/Applied-Biosystems.html?CID=fl-AppliedBiosystems) as described previously (Siddique *et al*., 2009). Each quantitative PCR sample contained 12.5 μl Platinum SYBR Green qPCR SuperMix with UDG (uracil DNA glycosylase) and ROX (5-carboxy-X-rhodamine) reference dye (Invitrogen), 2 mm MgCl_2_, 0.5 μl forward and reverse primer (10 μm), 2 μl cDNA and sufficient water to obtain a 25 μl total reaction volume. The primers used are shown in Table S2. All samples were diluted 1:3, and were analysed in technical triplicates.

### Nematode infection assays

*H. schachtii* cysts were harvested from *in vitro* stock cultures on roots of mustard plants (*Sinapis alba* cv. Albatros) growing on 0.2× Knop medium (Sijmons *et al*., 1991) supplemented with 2% sucrose. Hatching of J2 from the cysts was stimulated by soaking in 3 mm ZnCl_2_. Larvae were then washed three times in sterile water, and the J2 were resuspended in 0.5% w/v gelrite (Duchefa; http://www.duchefa-biochemie.nl/). Roots of 12-day-old Arabidopsis seedlings growing on Knop medium were inoculated under sterile conditions with approximately 50-60 J2 per plant. Three independent experiments were performed, each comprising five Petri dishes with a total of approximately ten seedlings. At 14 dpi, the size of syncytia (associated with females) and of females was measured as described by Siddique *et al*. (2009). Briefly, syncytia associated with females were randomly selected and photographed (longitudinal optical sections) using a Zeiss Axiovert 200M inverted microscope equipped with a Zeiss Axiocam digital camera (Zeiss, http://www.zeiss.com/). The syncytia and females were outlined using the Axiovision Kontour tool, and the area was determined. The mean size of syncytia and females was calculated, and data were further statistically analysed using single-factor anova (*P* < 0.05) and fisher's least significant difference test. One day later (15 dpi), the number of males and females was counted. The data were expressed as nematodes per cm of root length, and were analysed using single-factor anova (*P* < 0.05) and Duncan's multiple range test.

### GUS reporter analysis

The promoter region of *At1g64110* was amplified by PCR using 50 ng Arabidopsis genomic DNA as template. Primers (Table S2) included restriction sites for *Nco*I and *Eco*RI (underlined) for subsequent cloning into binary vector pPZP3425 (Szakasits *et al*., 2007). This plasmid contains a double enhanced 35S promoter and a TMV (tobacco mosaic virus) omega element driving the GUS gene. During the cloning procedure, the double enhanced 35S promoter and TMV omega element were replaced by the promoter fragment. The promoter sequence was confirmed by sequencing. We produced 17 GUS lines and tested seven in detail for GUS expression in seedlings and syncytia. From these, we selected the one line that was used here. Histochemical detection of GUS activity was performed by staining using X-Gluc (Biomol; http://www.biomol.de) in 0.1 m sodium phosphate buffer pH 7.0, 0.1% Triton X-100, 0.5 mm K_3_[Fe(CN)_6_], 0.5 mM K_4_[Fe(CN)_6_] and 10 mM Na_2_EDTA. After staining, chlorophyll was removed from photosynthetic tissues using 70% v/v ethanol. Various plant parts were stained after flowering of the plants. For seed imbibition, seeds were imbibed at 4°C on filter papers soaked in sterile water under continuous white light for 4 days before GUS staining for 24 h. For analysis of syncytia, infected roots containing syncytia were stained at various times after inoculation by overnight incubation with X-Gluc. The stained syncytia and uninfected roots were photographed under the Axiovert 200M inverted microscope with an integrated camera (AxioCam MRc5).

### *In vitro* pollen germination and GUS staining

For sperm cell staining, fresh anthers were used for *in vitro* pollen germination. The medium for *in vitro* pollen germination contained 18% sucrose, 0.01% boric acid, 1 mm CaCl_2_, 1 mm Ca(NO_3_)_2_, 1 mm MgSo_4_ and 0.5% agar (Difco; http://www.bd.com) at pH 7.0. Sucrose was dissolved first, and then other ingredients were added. Finally, agar was added and the medium was boiled for 3–4 min to dissolve agar (Li *et al*., 1999). The medium was poured into small Petri dishes (35 × 10 mm). Pollen was dusted onto the medium and allowed to germinate for 3–5 h or sometimes overnight at room temperature. After pollen germination, histochemical detection of GUS activity was performed by staining using the above-mentioned X-Gluc solution and incubation overnight at 37°C. The pollen tubes containing stained sperm cells were photographed as described above.

### *In situ* RT-PCR

*In situ* RT-PCR was performed as described by Koltai and Bird (2000) and Urbanczyk-Wochniak *et al*. (2002). Syncytia at 15 dpi were dissected from roots and immediately placed into cold fixation solution (63% v/v ethanol, 2% v/v formalin). After 24 h, syncytia were embedded in 4% low-melting-point agarose, and 25 μm thick sections were prepared using a vibratome (VT100; Leica, http://www.leica.com/). RT-PCR was then performed using digoxigenin-labelled dUTP and primers (Table S2). After a staining reaction with nitroblue tetrazolium/5-bromo-4-chloro-3-indolyl phosphate, cross-sections were photographed under an Axiovert 200M inverted microscope with an integrated camera (AxioCam MRc5). Full details of the method are provided in Wieczorek *et al*. (2006).

### Mutant screening

Seeds of SAIL_804_F09, SALK_023626 (*At1g64110*), SALK_150112 and SALK_146218 (*At5g52882*) were obtained from the Nottingham Arabidopsis Stock Centre (http://arabidopsis.info/) (Sessions *et al*., 2002; Alonso *et al*., 2003). Genomic DNA from segregating plants was screened by PCR using the primers listed in Table S2. Primer sequences were obtained using the tool supplied at the SIGnAL website (http://signal.salk.edu/tdnaprimers.2.html).

### Root length and area measurement

Seeds of Arabidopsis plants (Col-0 and T-DNA mutants) were grown on Knop medium as described above. Twelve-day-old plants (corresponding to the infection time point) were photographed using a DM4000 microscope (Leica; http://www.leica-microsystems.com) and an Olympus digital camera (http://www.olympus-europa.com/). The length and diameter of main and all lateral roots was calculated using the measurement tool of las software version 4.1 (http://www.leica-microsystems.com/products/microscope-software/materials-sciences/materials-imaging/details/product/leica-las-interactive-measurement/). The total root area of a plant was calculated by adding the areas of all individual roots. Five to ten individual measurements were used to calculate the mean length and area per plant.

### Bioinformatics

Sequence similarity searches indicated the presence of a conserved AAA+ domain in the C-terminal part of At1g64110. Homologues of this domain were identified by searching the National Center for Biotechnology Information non-redundant database using BLAST (Altschul *et al*., 1997). The 2000 most similar high-scoring segment pairs were retrieved and clustered using CLANS (Frickey and Lupas, 2004a), with BLAST as a comparison tool. Clustering was performed with default parameters at a *P* value cut-off of 1.0e-50. Searches using HHpred (Soding *et al*., 2005), a remote homology detection method based on comparison of profile hidden Markov models, revealed the presence of another but degenerate AAA+ domain in the N-terminal portion of At1g64110. The N-terminal transmembrane helix was assigned using TMHMM (Krogh *et al*., 2001).

## SUPPORTING INFORMATION

**Table S1.** Gene expression of ATPase genes in syncytia (from Szakasits *et al*., 2009).

**Table S2.** Primers used in this work.

**Table S3.** Expression of validated DUO1 target genes (Borg *et al*., 2011) in syncytia induced by *H. schachtii* in Arabidopsis roots.

**Table S4.** Expression of genes identified as ‘significantly differentially regulated according to both ATHK1 transcript level and sorbitol stress condition’ (Wohlbach *et al*., 2008) in syncytia induced by *H. schachtii* in Arabidopsis roots.

**Table S5.** Expression of genes up-regulated in plants over-expressing AREB1ΔQT (Fujita *et al*., 2005) in syncytia induced by *H. schachtii* in Arabidopsis roots.

**Figure S1.** Expression of *At1g64110* and *At1g52882* in syncytia as shown by RT-PCR.

**Figure S2.** Gene expression of *At1g64110* and *At4g28000* according to Genevestigator.

**Figure S3.** GUS expression in uninfected seedlings.

**Figure S4.** Correlation of syncytia size and female nematode size with the expression of *At1g64110* in miRNA lines with various promoters.

**Figure S5.** Phenotypic analysis of T-DNA mutants.

**Figure S6.** Sequence alignment of ORTHO000440 ATPases.

**Figure S7.** Domain composition of At1g64110.

**Methods S1.** Cloning of pUCmi319a.
