# Supplementary material for: An Arabidopsis ATPase gene involved in nematode-induced syncytium development and abiotic stress responses
Source: Plant J. 2013 Mar 8;74(5):852–66. doi: 10.1111/tpj.12170 (PMC3712482; doi:10.1111/tpj.12170)
Supplement: Supplementary file 1 [file tpj0074-0852-SD1.docx]

**Supplemental Figure S1.**

**
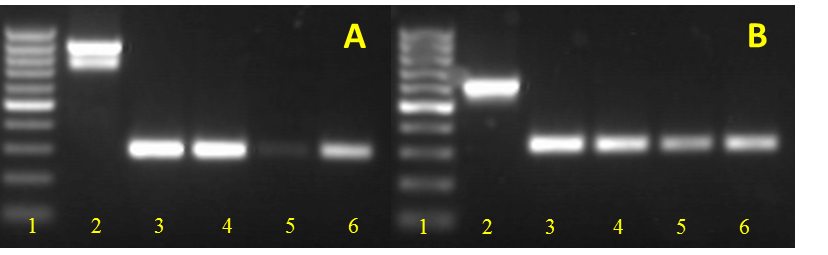
**

**Figure S1.** Expression of *At1g64110* (A) and *At1g52882* (B) in syncytia as shown by RT-PCR. 1, 100 bp ladder; 2, PCR with genomic DNA (expected size: 890 bp for *At1g64110* and 617 bp for *At1g52882*). 3-6, RT-PCR (expected size: 302 bp for *At1g64110* and 334 bp for *At1g52882*) with RNA from syncytia cut out from the roots and RNA from control root segments. 3, 5 dpi syncytia; 4, 15 dpi syncytia; 5, control segments from root elongation zone without root tip of 13 day old seedlings; 6, control segments from older and younger root parts without root tips of 13 day old seedlings.
