# Supplementary material for: An Arabidopsis ATPase gene involved in nematode-induced syncytium development and abiotic stress responses
Source: Plant J. 2013 Mar 8;74(5):852–66. doi: 10.1111/tpj.12170 (PMC3712482; doi:10.1111/tpj.12170)

**Supplemental Figure S2.** Gene expression of *At1g64110* and *At4g28000* according to Genevestigator (https://www.genevestigator.com/gv/).


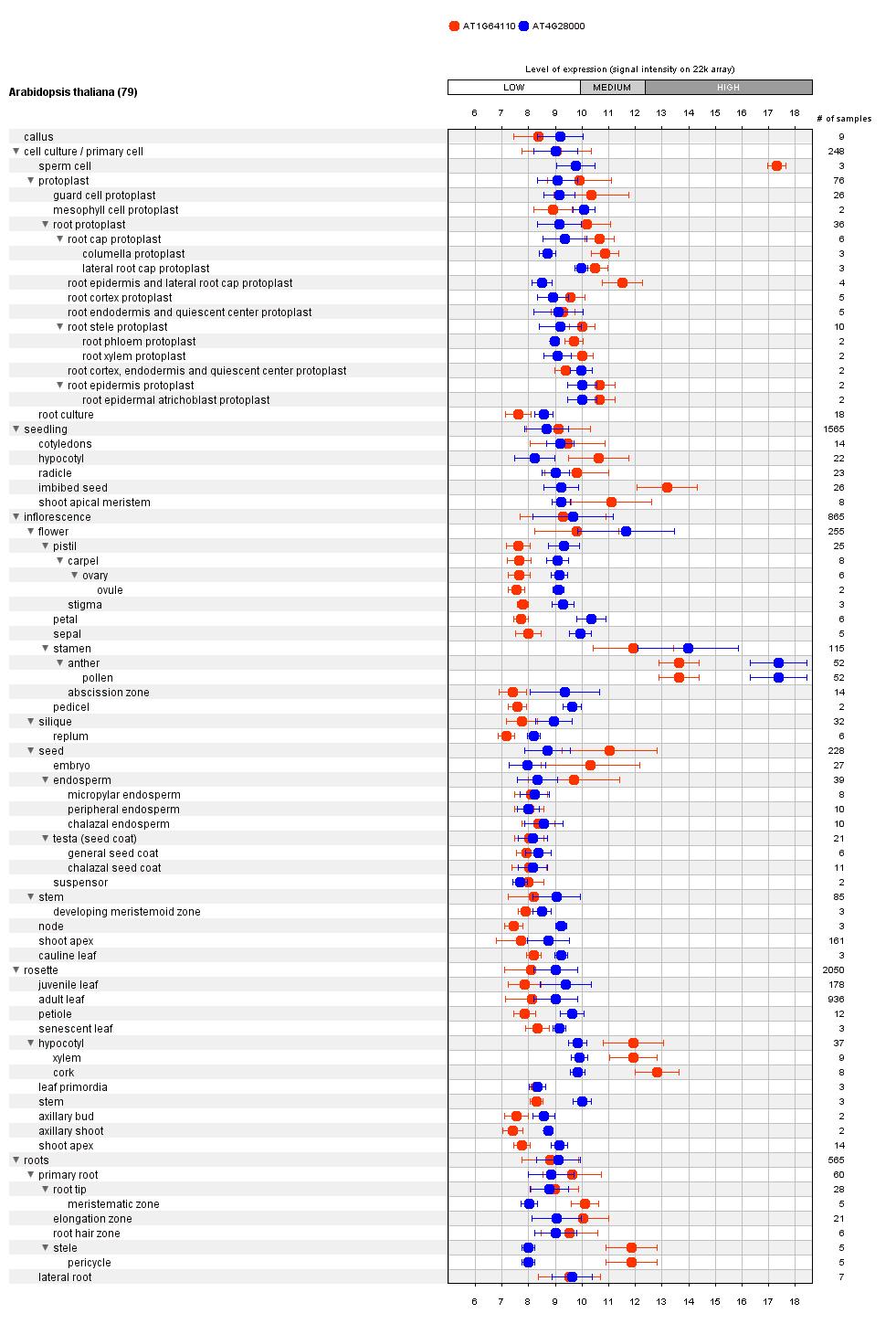

Supplement: Supplementary file 2 [file tpj0074-0852-SD2.docx]
