# Supplementary material for: An Arabidopsis ATPase gene involved in nematode-induced syncytium development and abiotic stress responses
Source: Plant J. 2013 Mar 8;74(5):852–66. doi: 10.1111/tpj.12170 (PMC3712482; doi:10.1111/tpj.12170)
Supplement: Supplementary file 3 [file tpj0074-0852-SD3.docx]

**Supplemental Figure S3.** GUS expression in uninfected seedlings


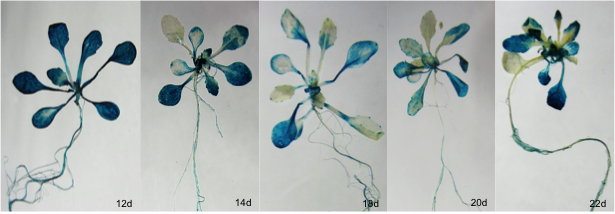

12 days old seedlings showed GUS staining both in the roots and the shoots. However, intensity of the staining decreased considerably in older leaves and older roots except for trichomes and staining at the edges of the leaves. In uninfected roots, staining was observed in the central cylinder (pericycle cells). Bar, 100 µm,
