# Supplementary material for: An Arabidopsis ATPase gene involved in nematode-induced syncytium development and abiotic stress responses
Source: Plant J. 2013 Mar 8;74(5):852–66. doi: 10.1111/tpj.12170 (PMC3712482; doi:10.1111/tpj.12170)

**Supplemental Figure S4.** Correlation of syncytia size and female nematode size with the expression of *At1g64110* in miRNA lines with different promoters.


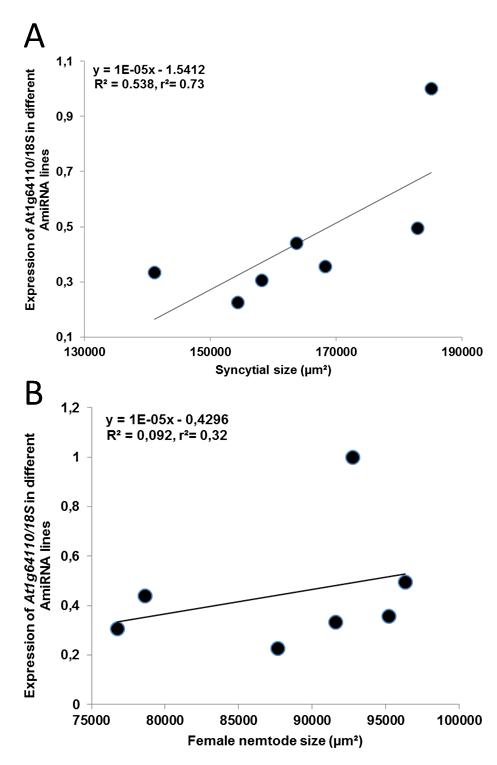

Supplement: Supplementary file 4 [file tpj0074-0852-SD4.docx]
