# Supplementary material for: An Arabidopsis ATPase gene involved in nematode-induced syncytium development and abiotic stress responses
Source: Plant J. 2013 Mar 8;74(5):852–66. doi: 10.1111/tpj.12170 (PMC3712482; doi:10.1111/tpj.12170)
Supplement: Supplementary file 5 [file tpj0074-0852-SD5.docx]

**Supplemental Figure S5.** Phenotypic analysis of T-DNA mutants

**
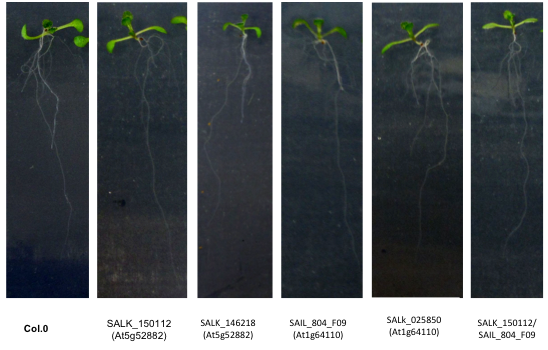
**

Length and area of root for 12 day old seedlings (corresponding to the time of nematode infection). There was a significant decrease in root area and length for SALK_146218 (*At5g52882*) as compared to Col wild type plants.
