# Supplementary figures and images for: An Arabidopsis ATPase gene involved in nematode-induced syncytium development and abiotic stress responses
Source: Plant J. 2013 Mar 8;74(5):852–66. doi: 10.1111/tpj.12170 (PMC3712482; doi:10.1111/tpj.12170)

**Supplemental Figure S6.** Sequence alignment of the three Arabidopsis ORTHO000440 ATPases.

**
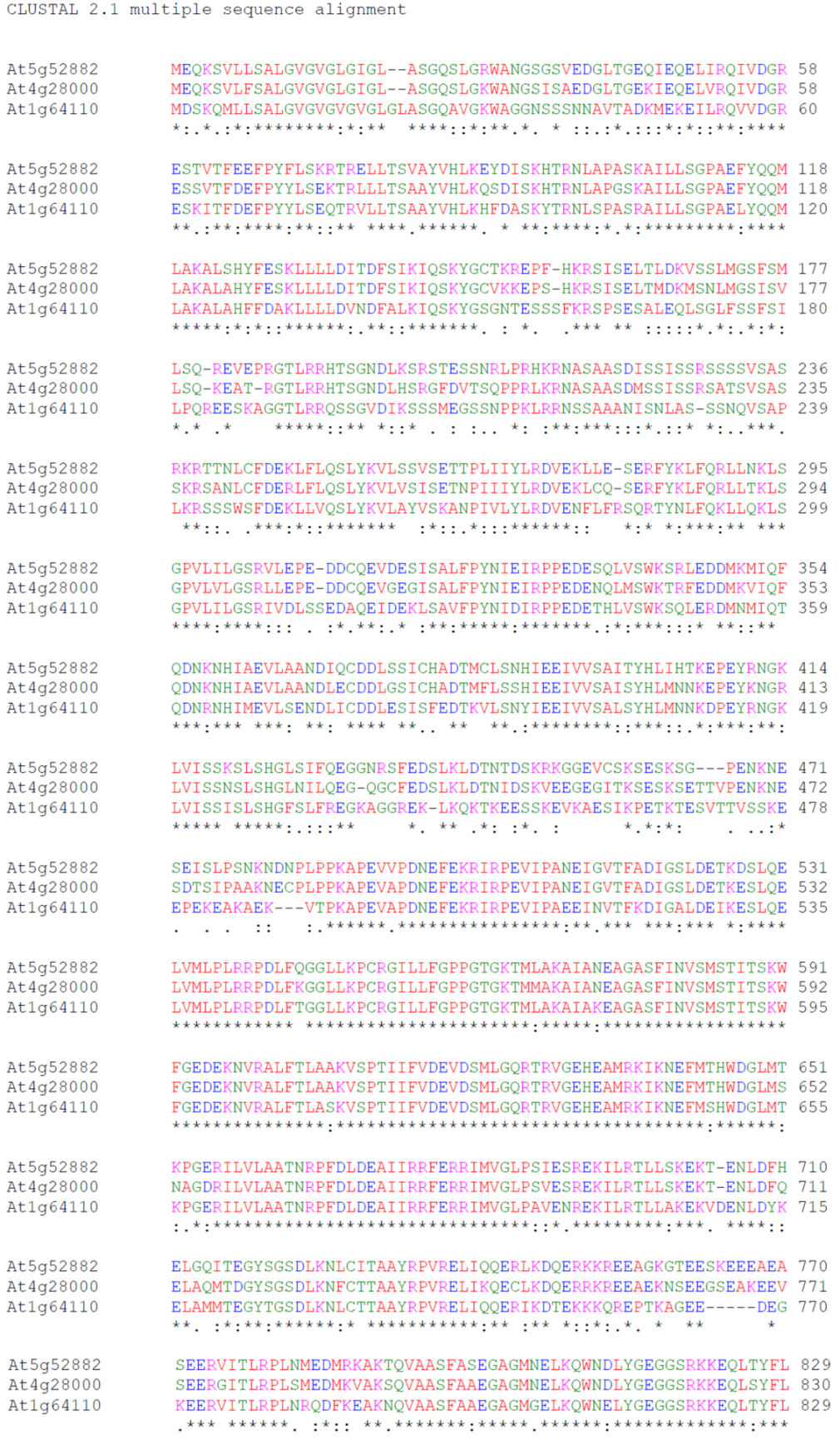
**

Supplement: Supplementary file 6 [file tpj0074-0852-SD6.docx]
