# Supplementary material for: An Arabidopsis ATPase gene involved in nematode-induced syncytium development and abiotic stress responses
Source: Plant J. 2013 Mar 8;74(5):852–66. doi: 10.1111/tpj.12170 (PMC3712482; doi:10.1111/tpj.12170)
Supplement: Supplementary file 7 [file tpj0074-0852-SD7.docx]

**Supplemental Figure S7.** Domain composition of At1G64110.

**
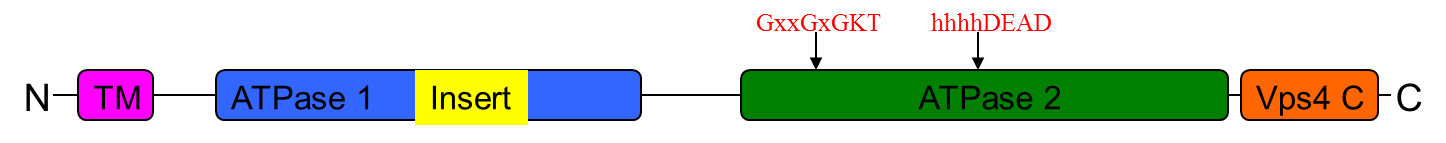
**

- Pink: transmembrane helix
- Blue: ATPase domain 1; no enzymatic activity, rather only a structural domain; Walker A nd B are degenerate
- Yellow: large insertion into the first ATPase domain
- Green: ATPase domain 2; funtional (canonical Walker A and B motifs in red)
- Orange: C-terminal extension similar to the one found in Vps4; presumably important for oligomerization
