# Supplementary material for: An Arabidopsis ATPase gene involved in nematode-induced syncytium development and abiotic stress responses
Source: Plant J. 2013 Mar 8;74(5):852–66. doi: 10.1111/tpj.12170 (PMC3712482; doi:10.1111/tpj.12170)
Supplement: Supplementary file 8 [file tpj0074-0852-SD8.docx]

**Table S1**

Gene expression of ATPase genes in syncytia induced by *H. schachtii* in Arabidopsis roots according to (Szakasits et al., 2009)

| **Gene ID** | **Syncytium** | **Root** | **M value** |
| --- | --- | --- | --- |
| ***At1g64110*** | **11.0** | **3.3** | **7.7*** |
| ***At4g28000*** | **3.5** | **3.4** | **0.1** |
| ***At5g52882*** | **n.a.** | **n.a.** | **n.a.** |

All values are in log2ratio.

*Indicates significant upregulation or downregulation (false discovery rate < 5%).
