# Supplementary material for: An Arabidopsis ATPase gene involved in nematode-induced syncytium development and abiotic stress responses
Source: Plant J. 2013 Mar 8;74(5):852–66. doi: 10.1111/tpj.12170 (PMC3712482; doi:10.1111/tpj.12170)
Supplement: Supplementary file 9 [file tpj0074-0852-SD9.docx]

**Table S2**

Primers used in this work (restriction sites are underlined)

| Primer | Sequence (5´-3´) | Amplification |
| --- | --- | --- |
| pAt1g64110Ecofor | TCTCTGAACGAATTCATTAGGAAGTAAC | *At1g64110* promoter fragment |
| pAt1g64110Ncorev | TGGACAGCAAACCCATGGTGTTGTCGG |  |
| At1g64110for | GTGGGTTTAGGCTTGGCTTCT | Real time *At1g64110* |
| At1g64110rev | TGTTGGTAAAGCTCGGCAGG |  |
| At4g28000F2 | TGAATGCCCCCTGCCTCCGA | Real time *At4g28000* |
| At4g28000R2 | CACTTTGGCCGCGAGGGTGA |  |
| At5g52882for2 | GAGCTTGGGCAGATAACAGA | Real time *At5g52882* |
| At5g52882for2 | TTTTCCTTGAGCCTCCTTCT |  |
| At1g64110for | GTGGGTTTAGGCTTGGCTTCT | RT-PCR *At1g64110* |
| At1g64110rev | TGTTGGTAAAGCTCGGCAGG |  |
| At4g28000F2 | TGAATGCCCCCTGCCTCCGA | RT-PCR *At4g28000* |
| At4g28000R2 | CACTTTGGCCGCGAGGGTGA |  |
| At5g52882for2 | GAGCTTGGGCAGATAACAGA | RT-PCR *At5g52882* |
| At5g52882rev2 | TTTTCCTTGAGCCTCCTTCT |  |
| miRNA319for | CATAAGTCGACCATGGacaaacacacgctcggacgca | Amplification of miRNA319a gene from genomic DNA |
| miRNA319rev2 | GTATTGGATCCTcatggcgagccttaaataaag |  |
| Oligo Primer I | gaTAAACGTTTATGAAACTCGCCtctctcttttgtattcc | Cloning of amiRNA for At1g64110 |
| Oligo Primer II | gaGGCGAGTTTCATAAACGTTTAtcaaagagaatcaatga |  |
| Oligo Primer III | gaGGAGAGTTTCATATACGTTTTtcacaggtcgtgatatg |  |
| Oligo Primer IV | gaAAAACGTATATGAAACTCTCCtctacatatatattcct |  |
| Oligonucleotide A | CTGCAAGGCGATTAAGTTGGGTAAC |  |
| Oligonucleotide B | GCGGATAACAATTTCACACAGGAAACAG |  |
