# Supplementary material for: An Arabidopsis ATPase gene involved in nematode-induced syncytium development and abiotic stress responses
Source: Plant J. 2013 Mar 8;74(5):852–66. doi: 10.1111/tpj.12170 (PMC3712482; doi:10.1111/tpj.12170)
Supplement: Supplementary file 10 [file tpj0074-0852-SD10.docx]

**Table S3**

Expression of validated DUO1 target genes (Borg et al. 2011) in syncytia induced by *H. schachtii* in Arabidopsis roots

| Gene ID | Gene | Syncytium^1^ | Root^1^ |
| --- | --- | --- | --- |
| At1g19890 | MGH3/HTR10b | 2.4 | 2.3 |
| At2g17180 | DAZ1 | 2.6 | 2.5 |
| At3g04620 | DAN1 | 5.8* | 3.0 |
| At3g47440 | TIP5;1 | 3.4 | 3.7 |
| At3g62230 | DAF1 | 2.7 | 2.7 |
| At4g35280 | DAZ2 | 3.6 | 3.5 |
| At5g39650 | DAU2 | 2.3 | 2.3 |
| At5g49150 | GEX2b | 2.7 | 3.0 |
| At5g52000 | IMPa-8 | 2.8 | 3.0 |
| At5g53520 | OPT8 | 2.6 | 2.7 |
| At1g64110 | DAA1 | 11.0* | 3.3 |
| At1g68610 | PCR11 | 2.7 | 2.9 |
| At3g50310 | MAPKKK20 | 3.9 | 3.9 |
| At4g35560 | DAW1 | 5.4 | 8.1* |
| At4g35700 | DAZ3 | 2.8 | 3.0 |
| At4g11720 | GCS1/HAP2b | 2.7 | 2.5 |

^1^Data from Szakasits et al. (2009).

*indicates significant upregulation (green) or downregulation (red) (false discovery rate < 5%).

*At1g64110*/*DAA1* marked yellow,
