# Supplementary material for: An Arabidopsis ATPase gene involved in nematode-induced syncytium development and abiotic stress responses
Source: Plant J. 2013 Mar 8;74(5):852–66. doi: 10.1111/tpj.12170 (PMC3712482; doi:10.1111/tpj.12170)
Supplement: Supplementary file 11 [file tpj0074-0852-SD11.docx]

**Table S4**

Expression of genes identified as “Significantly Differentially Regulated According to Both ATHK1 Transcript Level and Sorbitol Stress Condition” (Wohlbach et al. 2008) in syncytia induced by *H. schachtii* in Arabidopsis roots.

| Gene ID | Function | Syncytium^1^ | Root^1^ |
| --- | --- | --- | --- |
| At2g17820 | AtHK1 | 4.3 | 4.8 |
|  |  |  |  |
| At2g39800 | P5CS1d 1-pyrroline-5-carboxylate synthetase | 3.8 | 4.4 |
| At4g02280 | Sucrose synthase | 5.4* | 4.2 |
| At3g60140 | DIN2 glycosyl hydrolase family 1 protein | 5.8* | 3.2 |
| At5g43840 | AT-HSFA6A heat shock transcription factor | 3.1 | 3.3 |
| At3g61890 | ATHB-12 homeobox-leucine zipper | 4.0 | 3.4 |
| At4g19170 | NCED4 9-cis-epoxycarotenoid dioxygenase, putative | 3.6 | 3.3 |
| At3g13784 | b-Fructosidase, putative | 3.3 | 3.1 |
| At5g20830 | SUS1 sucrose synthase | 6.3 | 6.8 |
| At2g46680 | ATHB-7 similar to homeobox-leucine zipper | 6.7 | 7.7 |
| At1g17870 | S2P-like putative metalloprotease | 4.6 | 4.3 |
| At1g73480 | Hydrolase, a/b fold family protein | 4.2 | 3.8 |
| At2g41190 | Amino acid transporter family protein | 6.2 | 5.2 |
| At2g33380 | RD20 similar to Ca2þ binding EF hand | 2.3 | 2.4 |
| At5g52300 | RD29B stress-responsive protein related | 6.7* | 5.2 |
| At3g02480 | ABA-responsive protein related | 8.6* | 3.2 |
| At1g64110 | AAA-type ATPase family protein | 11.0* | 3.3 |
| At1g53540 | 17.6-kD class I small heat shock protein | 5.4 | 5.3 |
| At1g07430 | Protein phosphatase 2C, putative | 8.8* | 4.2 |
| At1g62510 | Protease inhibitor/seed storage/lipid transfer protein | 1.9 | 2.8 |
| At5g12030 | AT-HSP17.6A 1 class II heat shock protein | 4.0 | 3.8 |
| At4g26790 | GDSL-motif lipase/hydrolase | 2.7 | 3.8* |
| At2g21320 | Zinc finger (B-box type) | 6.8* | 4.0 |
| At3g62590 | Lipase class | 5.5 | 5.1 |
| At3g09270 | ATGSTU8 glutathione S-transferase, putative | 5.3 | 8.6* |
| At1g80820 | CCR2 cinnamoyl-CoA reductase, putative | 9.1* | 3.4 |
| At3g09640 | APX2 similar to L-ascorbate peroxidase | 3.1 | 3.0 |
| At2g19900 | ATNADP-ME1 malic enzyme | 6.0* | 4.7 |
| At5g53870 | Plastocyanin-like domain-containing protein | 2.2 | 2.2 |
| At2g46270 | GBF3 G-box binding factor | 5.2 | 5.2 |
| At1g68570 | Proton-dependent oligopeptide transport | 3.2 | 3.4 |
| At3g46230 | ATHSP17.4 class I heat shock protein | 5.8* | 4.8 |
| At5g06530 | Similar to ABC transporter family protein | 3.2 | 3.5 |
| At4g10250 | ATHSP22.0 ER small heat shock protein | 3.2* | 2.6 |
| At5g37500 | GORK guard cell outward rectifying Kþ channel | 3.5 | 5.2* |
| At3g28270 | Expressed protein | 2.7 | 2.8 |
| At5g09930 | ATGCN2 ABC transporter family protein | 2.8 | 2.7 |
| At2g04160 | AIR3 subtilisin-like protease | 2.9 | 3.1 |
| At3g62740 | Glycosyl hydrolase family 1 protein | 3.0 | 3.1 |

^1^Data from Szakasits et al. (2009),

*indicates significant upregulation (green) or downregulation (red) (false discovery rate < 5%).

*At1g64110*/*DAA1* marked yellow,
