# Supplementary material for: An Arabidopsis ATPase gene involved in nematode-induced syncytium development and abiotic stress responses
Source: Plant J. 2013 Mar 8;74(5):852–66. doi: 10.1111/tpj.12170 (PMC3712482; doi:10.1111/tpj.12170)
Supplement: Supplementary file 12 [file tpj0074-0852-SD12.docx]

**Table S5**

Expression of genes upregulated in plants overexpressing AREB1ΔQT (Fujita et al. 2005) in syncytia induced by *H. schachtii* in Arabidopsis roots.

| Gene ID | Gene Symbol | Syncytium^1^ | Root^1^ |
| --- | --- | --- | --- |
| At1g45249 | AREB1/ABF2 | - | - |
| At3g19290 | AREB2/ABF4 | 4.6 | 6.6* |
| At3g56850 | AREB3/DPBF3 | 6.0 | 5.6 |
| At1g49720 | ABF1 | 5.0 | 6.3* |
| At4g34000 | ABF3/DPBF5 | 4.4 | 7.1* |
| At2g36270 | ABI5/DPBF1 | 5.2 | 4.7 |
| At2g41070 | EEL/DPBF4 | 2.9 | 3.0 |
| At3g44460 | DPBF2 | 2.6 | 2.7 |
| At2g18050 | HIS1-3 | 4.9* | 2.8 |
| At1g64110 | (AIA1) | 11.0* | 3.3 |
| At3g17520 | AIL1 | 4.4 | 3.6 |
| At5g42800 | Dihydroflavonol  4-reductase | 3.0 | 3.0 |
| At5g52300 | RD29B | 6.7* | 5.2 |
| At2g46270 | GBF3 | 5.2 | 5.2 |
| At2g33380 | RD20 | 2.3 | 2.4 |
| At5g66400 | RAB18 | 5.1 | 4.5 |

^1^Data from Szakasits et al. (2009),

*indicates significant upregulation (green) or downregulation (red) (false discovery rate < 5%).

*At1g64110*/*DAA1* marked yellow,
