# Supplementary material for: An Arabidopsis ATPase gene involved in nematode-induced syncytium development and abiotic stress responses
Source: Plant J. 2013 Mar 8;74(5):852–66. doi: 10.1111/tpj.12170 (PMC3712482; doi:10.1111/tpj.12170)
Supplement: Supplementary file 13 [file tpj0074-0852-SD13.docx]

**SUPPLEMENTARY MATERIAL**

**Supplemental Methods**

**Cloning of pUCmi319a**

The Arabidopsis miRNA319a gene was amplified by PCR with primers miRNA319for (containing and a SalI site for cloning and introducing a NcoI site) and miRNA319rev2 (containing a BamHI site) using genomic Arabidopsis DNA as template. The PCR product was digested with SalI and BamHI and cloned into pUC18 (Yanisch-Perron *et al.* 1985) digested with the same enzymes. The correct sequence of pUCmi319a was confirmed by sequencing.

**BACKBONE miRNA319:**

acaaacacacgctcggacgcatattacacatgttcatacacttaatactcgctgttttgaattgatgttttaggaatatatatgtaga**GAGAGCTTCCTTGAGTCCAT**TCACAGGTCGTGATATGATTCAATTAGCTTCCGACTCATTCATCCAAATACCGAGTCGCCAAAATTCAAACTAGACTCGTTAAATGAATGAATGATGCGGTAGACAAATTGGATCATTGATTCTCTTTGA**TTGGACTGAAGGGAGCTCCC**tctctcttttgtattccaattttcttgattaatctttcctgcacaaaaacatgcttgatccactaagtgacatatatgctgccttcgtatatatagttctggtaaaattaacattttgggtttatctttatttaaggcatcgccatg
