# Supplementary material for: An Arabidopsis ATPase gene involved in nematode-induced syncytium development and abiotic stress responses
Source: Plant J. 2013 Mar 8;74(5):852–66. doi: 10.1111/tpj.12170 (PMC3712482; doi:10.1111/tpj.12170)
Supplement: Supplementary file 14 [file tpj0074-0852-SD14.docx]

**Supplemental Material**

**Supplemental Methods.** Cloning of pUCmi319a.

**Supplemental Table S1.** Gene expression of ATPase genes in syncytia (according to Szakasits *et al.* 2009).

**Supplemental Table S2.** Primers used in this work.

**Supplemental Table S3.** Expression of validated DUO1 target genes (Borg et al. 2011) in syncytia induced by *H. schachtii* in Arabidopsis roots.

**Supplemental Table S4.** Expression of genes identified as “Significantly Differentially Regulated According to Both ATHK1 Transcript Level and Sorbitol Stress Condition” (Wohlbach et al. 2008) in syncytia induced by *H. schachtii* in Arabidopsis roots.

**Supplemental Table S5.** Expression of genes upregulated in plants overexpressing AREB1ΔQT (Fujita et al. 2005) in syncytia induced by *H. schachtii* in Arabidopsis roots.

**Supplemental Figure S1.** Expression of *At1g64110* and *At1g52882* in syncytia as shown by RT-PCR.

**Supplemental Figure S2.** Gene expression of *At1g64110* and *At4g28000* according to Genevestigator.

**Supplemental Figure S3.** GUS expression in uninfected seedlings

**Supplemental Figure S4.** Correlation of syncytia size and female nematode size with the expression of *At1g64110* in miRNA lines with different promoters.

**Supplemental Figure S5.** Phenotypic analysis of T-DNA mutants

**Supplemental Figure S6.** Sequence alignment of ORTHO000440 ATPases.

**Supplemental Figure S7.** Domain composition of At1g64110.

**Table S1**

Gene expression of ATPase genes in syncytia induced by *H. schachtii* in Arabidopsis roots according to (Szakasits et al., 2009)

All values are in log2ratio.

*Indicates significant upregulation or downregulation (false discovery rate < 5%).

**Table S2**

Primers used in this work (restriction sites are underlined)

**Table S3**

Expression of validated DUO1 target genes (Borg et al. 2011) in syncytia induced by *H. schachtii* in Arabidopsis roots

^1^Data from Szakasits et al. (2009)

*indicates significant upregulation (green) or downregulation (red) (false discovery rate < 5%).

*At1g64110*/*DAA1* marked yellow

**Table S4**

Expression of genes identified as “Significantly Differentially Regulated According to Both ATHK1 Transcript Level and Sorbitol Stress Condition” (Wohlbach et al. 2008) in syncytia induced by *H. schachtii* in Arabidopsis roots.

^1^Data from Szakasits et al. (2009),

*indicates significant upregulation (green) or downregulation (red) (false discovery rate < 5%).

*At1g64110*/*DAA1* marked yellow,

**Table S5**

Expression of genes upregulated in plants overexpressing AREB1ΔQT (Fujita et al. 2005) in syncytia induced by *H. schachtii* in Arabidopsis roots.

^1^Data from Szakasits et al. (2009),

*indicates significant upregulation (green) or downregulation (red) (false discovery rate < 5%).

*At1g64110*/*DAA1* marked yellow,

**Figure S1.** Expression of *At1g64110* (A) and *At1g52882* (B) in syncytia as shown by RT-PCR. 1, 100 bp ladder; 2, PCR with genomic DNA (expected size: 890 bp for *At1g64110* and 617 bp for *At1g52882*). 3-6, RT-PCR (expected size: 302 bp for *At1g64110* and 334 bp for *At1g52882*) with RNA from syncytia cut out from the roots and RNA from control root segments. 3, 5 dpi syncytia; 4, 15 dpi syncytia; 5, control segments from root elongation zone without root tip of 13 day old seedlings; 6, control segments from older and younger root parts without root tips of 13 day old seedlings.

**Supplemental Figure S2.** Gene expression of *At1g64110* and *At4g28000* according to Genevestigator (https://www.genevestigator.com/gv/).

**Supplemental Figure S3.** GUS expression in uninfected seedlings

12 days old seedlings showed GUS staining both in the roots and the shoots. However, intensity of the staining decreased considerably in older leaves and older roots except for trichomes and staining at the edges of the leaves. In uninfected roots, staining was observed in the central cylinder (pericycle cells). Bar, 100 µm,

**Supplemental Figure S4.** Correlation of syncytia size and female nematode size with the expression of *At1g64110* in miRNA lines with different promoters.

**Supplemental Figure S5.** Phenotypic analysis of T-DNA mutants

Length and area of root for 12 day old seedlings (corresponding to the time of nematode infection). There was a significant decrease in root area and length for SALK_146218 (*At5g52882*) as compared to Col wild type plants.

**Supplemental Figure S6.** Sequence alignment of the three Arabidopsis ORTHO000440 ATPases.

**Supplemental Figure S7.** Domain composition of At1G64110.

- Pink: transmembrane helix
- Blue: ATPase domain 1; no enzymatic activity, rather only a structural domain; Walker A nd B are degenerate
- Yellow: large insertion into the first ATPase domain
- Green: ATPase domain 2; funtional (canonical Walker A and B motifs in red)
- Orange: C-terminal extension similar to the one found in Vps4; presumably important for oligomerization
